# Targeted FMD Vaccines for Eastern Africa: The AgResults Foot and Mouth Disease Vaccine Challenge Project

**DOI:** 10.3390/v13091830

**Published:** 2021-09-14

**Authors:** Jef M. Hammond, Badi Maulidi, Nina Henning

**Affiliations:** Global Alliance for Livestock Veterinary Medicines (GALVmed), Doherty Building, Pentlands Science Park, Bush Loan, Edinburgh EH26 0PZ, UK; badi.maulidi@galvmed.org (B.M.); Nina.Henning@galvmed.org (N.H.)

**Keywords:** foot and mouth disease, vaccines, FMD

## Abstract

As one of the most infectious livestock diseases in the world, foot and mouth disease (FMD) presents a constant global threat to animal trade and national economies. FMD remains a severe constraint on development and poverty reduction throughout the developing world due to many reasons, including the cost of control measures, closure of access to valuable global FMD-free markets for livestock products, production losses through reduced milk yield, reduced live weight gain, and the inability of infected livestock to perform traction. FMD virus infects a variety of cloven-hoofed animals, including cattle, sheep, goats, swine, all wild ruminants, and suidae, with high morbidity in adult animals. High mortality can occur in young animals due to myocarditis. FMD is endemic in Africa, most of Asia, the Middle East, and parts of South America. The global clustering of FMD viruses has been divided into seven virus pools, where multiple serotypes occur but within which are topotypes that remain mostly confined to that pool. Three pools cover Europe, the Middle East, and Asia; three pools cover Africa; and one pool covers the Americas. The highly infectious nature of FMDV, the existence of numerous continually circulating serotypes and associated topotypes, the potential for wildlife reservoirs, and the frequent emergence of new strains that are poorly matched to existing vaccines all serve to compound the difficulties faced by the governments of endemic countries to effectively control and reduce the burden of the disease at the national and regional levels. This clustering of viruses suggests that if vaccination is to be a major tool for control, each pool could benefit from the use of tailored or more specific vaccines relevant to the topotypes present in that pool, rather than a continued reliance on the currently more widely available vaccines. It should also be noted that, currently, there are varying degrees of effort to identify improved vaccines in different regions. There are relatively few targeted for use in Africa, while the developed world’s vaccine banks have a good stock of vaccines destined for emergency outbreak use in FMDV-free countries. The AgResults Foot and Mouth Disease (FMD) Vaccine Challenge Project (the “Project”) is an eight-year, US $17.68 million prize competition that supports the development and uptake of high-quality quadrivalent FMD vaccines tailored to meet the needs of Eastern Africa (EA). The Project targets the following Pool Four countries: Burundi, Ethiopia, Kenya, Rwanda, Tanzania and Uganda. The Project is being run in two phases: a development phase, which will encourage the production of regionally relevant vaccines, and a cost-share phase, designed to help to reduce the price of these vaccines in the marketplace to the end users, which is hoped will encourage broader uptake. Manufacturers can submit quadrivalent FMD vaccines containing serotypes A, O, SAT1, and SAT2, which will be assessed as relevant for use in the region through a unique component of the Project requiring the screening of vaccines against the Eastern Africa Foot and Mouth Disease Virus Reference Antigen Panel assembled by the World Reference Laboratory for FMD (WRLFMD), at the Pirbright Institute, UK, in collaboration with the OIE/FAO FMD Reference Laboratory Network. To be eligible for the Project, sera from vaccinated cattle will be used to evaluate serological responses of FMD vaccines for their suitability for use in Eastern African countries. If they pass a determined cut-off threshold, they will be confirmed as relevant for use in the region and will be entered into the Project’s cost-share phase.

## 1. Introduction

**Figure 1:**
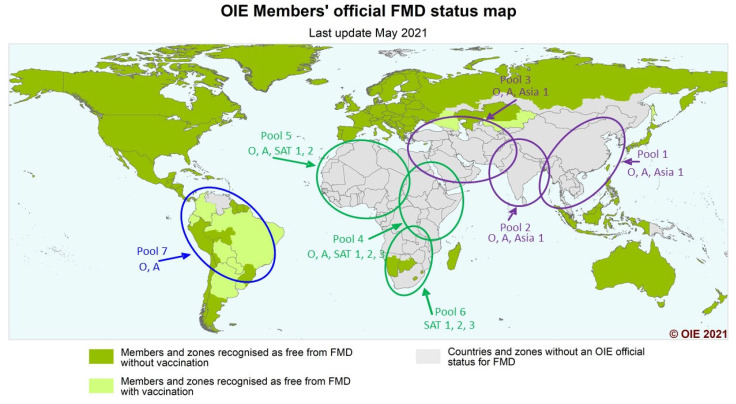
Global FMD situation and positions of virus pools. https://www.oie.int/app/uploads/2021/05/fmd-world-eng.png (accessed on 13 September 2021). Source: World Organisation for Animal Health (OIE). Reproduced with permission.

FMD virus is highly contagious, infecting a variety of cloven-hoofed animals, including cattle, sheep, goats, swine, all wild ruminants, and suidae [1,2,3]. The morbidity of FMD is high in infected adult livestock, but the disease is rarely fatal in adult animals. In contrast, high mortality can occur in young animals due to myocarditis. Following infection, the incubation period can be from 2 to 21 days, depending upon species, infectious dose, serotype, and strain, with an average of 3 to 8 days [2,3]. Large amounts of virus can be excreted by infected animals before clinical signs are evident [4]. Infected animals can exhibit blisters and ulcers on the mouth, tongue, lips, feet, and udder; lose weight; and stop producing milk, resulting in severe production losses that have been estimated to cost the African economy more than $2 billion annually [5]. Movement of live animals and animal products constitute the greatest risk for spread of FMD [6], and in parts of Africa, the Cape buffalo provides an important reservoir for the maintenance of certain FMD virus serotypes [7]. The virus exists as seven serotypes and numerous sub-lineages or topotypes and, in the field, FMDV continues to evolve, giving rise to new strains that cause periodic upsurges in the number of cases and increase the risk of spread into new areas. There is a global clustering of FMD viruses and these have been divided into seven virus pools, where multiple serotypes occur, but within which are topotypes or lineages that remain mostly confined to that pool. Three pools cover Europe, the Middle East and Asia; three pools cover Africa; and one pool covers the Americas (Figure 1) [7,8,9]. This enables global control of FMD to be implemented at a regional level. An increased regional knowledge of FMD outbreaks and identification of these within particular reservoirs or pools of FMD activity can greatly assist globally informed regional FMD control programmes. This approach also allows for the rapid identification of the movement of viruses out of their local pool and insurgence into other pools. With vaccination used as a major tool for control in endemic areas, each pool can benefit from investigation into the use of tailored or more specific vaccines relevant to the topotypes present in that pool, rather than a continued reliance on currently more widely available generic vaccines [10].

Increased global trade and movement of people and animals, both legal and illegal, provide opportunities for the virus to spread. The threat of FMD provides reason to restrict trade in animal products from affected countries to those without FMD, and thereby denies access of developing economies to the rich markets of the developed world, reducing incentives to improve productivity and efficiency [11]. Vaccines currently available for FMD control are based on preparations of the whole virus that are derived from cell cultures, chemically inactivated, and blended with suitable adjuvant. As the disease can be caused by seven different serotypes of virus, it is often necessary to include a combination of strains in the vaccine used to ensure protection. This is further complicated by the evolution of new subtypes of the virus. The more virus serotypes in a vaccine, the more expensive it becomes, restricting use in many developing countries [11]. There is also an absolute requirement for a cold chain, making widespread vaccination in developing countries a complex logistical challenge. The systemic use of FMD vaccines and the application of additional biosecurity measures effectively eradicated the disease from Europe [12], however, in many endemic settings, FMD vaccines are still primarily used reactively in response to an outbreak and without a proper control programme or follow-up procedures in place. The highly infectious nature of FMDV, the existence of numerous continually circulating serotypes and associated topotypes, the potential for wildlife reservoirs, and the frequent emergence of new strains that are poorly matched to existing vaccines all serve to compound the difficulties faced by the governments of endemic countries to effectively control and reduce the burden of the disease regionally. More recently, the adoption of the OIE/FAO Progressive Control Pathway for Foot and Mouth Disease control (PCP-FMD) [13,14] is providing endemic countries with a mechanism to initiate an FMD control plan. In addition, the use of high potency FMD vaccines in endemic settings has been suggested as a way to better control field viruses that may differ significantly from the available generic vaccine strains [10]. Importantly, successful control of FMD absolutely requires the availability of quality vaccines, but cannot be managed by vaccination alone, and there is a parallel need for improved surveillance through adequately resourced veterinary services, education of livestock keepers, rapid reporting and accurate real-time diagnosis of outbreaks and effective quarantine, disinfection of infected premises, and strict animal movement controls.

In an effort to improve the ability to select relevant vaccine strains to be used in the African region, a recently established OIE Laboratory Twinning project between WRLFMD at the Pirbright Institute, UK, and The African Union Pan African Veterinary Vaccine Centre (AU-PANVAC), Ethiopia, is developing an independent base to evaluate the quality of FMD vaccines in Africa. The main activities will include preparation and collation of reference materials, validation of methodologies, and technology transfer supported by training missions. Once fully established, AU-PANVAC will be able to carry out independent assessment of the quality of FMD vaccines intended for use in Africa, which will provide direct vaccine quality information to those responsible for vaccine purchase, and so help to improve the suitability and expected efficacy of vaccines used in the region.

## 2. The AgResults FMD Vaccine Challenge Project

The AgResults Initiative (“AgResults”) is a US $152 million multilateral programme financed jointly by the governments of Australia, Canada, the United Kingdom, the United States, and the Bill and Melinda Gates Foundation that uses pay-for-results prize competitions to incentivize, or “pull”, the private sector to overcome agricultural market barriers by investing in innovative research and delivery solutions that improve the lives of smallholder farmers. In doing so, AgResults goes beyond traditional “push”, or upfront grant funding by harnessing private sector competition and innovation in spurring sustained market improvement. AgResults has now launched their first livestock vaccine project for Eastern Africa, the “AgResults Foot and Mouth Disease Vaccine Challenge Project” (https://agresults.org/projects/fmd-vaccine, accessed on 13 September 2021). The AgResults Foot and Mouth Disease (FMD) Vaccine Challenge Project (the “Project”) is an eight-year, US $17.68 million prize competition that supports the development and uptake of high-quality FMD vaccines tailored to meet the needs of Eastern Africa (EA), targeting the following Pool 4 countries: Burundi, Ethiopia, Kenya, Rwanda, Tanzania, and Uganda. The Project is managed by The Global Alliance for Livestock Veterinary Medicines (GALVmed).

## 3. A Two-Phased Approach

The AgResults FMD Vaccine Challenge Project is being run in two phases: a development phase and a cost-share phase.

During the development phase, animal vaccine manufacturers will work on the development of FMD vaccines tailored to the needs of Eastern Africa (Pool 4). The target product profile (TPP) (Table 1.) set out in the competition rules (https://www.galvmed.org/wp-content/uploads/2021/03/FMD-Vaccine-Challenge-Project-RFA-FINAL-rev2-100321.pdf, accessed on 13 September 2021), defines the specific characteristics that a vaccine must satisfy, including standards related to safety, efficacy, and utility in the smallholder farmer setting. The Project also requires vaccines to be registered in at least two of the Project’s target countries—either through the East African Community Mutual Recognition Procedure (EAC MRP) or individual country registration procedures.

To be eligible for the cost-share phase, a manufacturer must submit an application to GALVmed demonstrating that its FMD vaccine meets all of the eligibility criteria listed in the TPP. Once an application has been approved by the Judging Panel, consisting of FMD, industry, and regulatory experts, the vaccine can then enter the cost-share phase.

During the cost-share phase, AgResults commits to funding a portion of the purchase price of the vaccine for a specified volume of vaccine doses and will provide funding directly to the vaccine manufacturers. This cost-share mechanism has been developed by AgResults to reduce the cost-per-dose for buyers who purchase directly from the approved vaccine manufacturer and thereby promote and support the adoption of new high-quality vaccines in the region. This will enable public and private sector actors to be better able to combat FMD through more consistent purchases of the new vaccines and will also increase the market potential for vaccines in the region. The cost-share awards will be available for four and a half years after the commencement of the cost-share phase.

Through this pay-for-results mechanism, the Project aims to achieve three objectives:

Development of high-quality FMD vaccines, tailored for Eastern African FMDV strains.

Increased FMD vaccine production and regional purchases to create greater market stability and reduce price.

Development of a private sector model for buying and distributing FMD vaccines to complement public sector efforts.

## 4. Current FMD Status in Target Countries (Information Taken from OIE/FAO FMD Reference Laboratory Network Annual Report 2019)

Serotypes O, A, SAT 1, and SAT 2 commonly circulate in Pool 4 of the EA region. The OIE/FAO FMD Reference Laboratory Network has reported that there are two separate sub-regional pools of endemic virus circulation within this pool [15]. FMDV topotype O/EA-3 is present in countries in the northern part of EA, including Ethiopia and Sudan, and a second topotype (O/EA-4) is present in Ethiopia. In contrast, countries in the southern part of the EA region have different serotype O topotypes, including O/EA-2, with a wide distribution in Kenya, Uganda, Tanzania, and Zambia, and O/EA-1, which has only been reported in Kenya and was last detected in 2009. This observed segregation between viruses found in the northern and southern parts of the EA region also appears to be repeated for the serotypes A, SAT 1, and SAT 2. In recent years, there has been a lack of detailed information on the real-time circulation of FMD viral lineages in the EA region, leading to the recommendations that FMD surveillance and characterisation capacity in the region should be improved with the implementation of regional control measures, improvements in the serological diagnostic test performance and laboratory capacity of the National Reference Laboratories (NRLs), and training of personnel especially strengthening the molecular diagnostic capacity [16]. This lack of available capacity can lead to under reporting of outbreaks and correspondingly low numbers of field samples collected and analysed by FMD reference laboratories. However, the most recent estimations of circulating FMD viral lineages in the competition target countries taken from the WRLFMD Country Reports for the Last 5 Years are shown in Table 2.

## 5. Improving the Regional Relevance of FMD Vaccines

The AgResults FMD Vaccine Challenge Project requires the development of quadrivalent FMD vaccines containing serotypes O, A, SAT1, and SAT2. Each vaccine submitted to the Project will be assessed as relevant for use in the region through a unique method that requires the screening of vaccines against the Eastern Africa Foot and Mouth Disease Virus Reference Antigen Panel (EA FMDV Reference Antigen Panel (https://www.wrlfmd.org/node/2096/, accessed on 13 September 2021). This antigen panel has been assembled by the WRLFMD, TPI, UK, in collaboration with the OIE/FAO FMD Reference Laboratory Network and comprises sixteen FMDV isolates representative of viruses currently predicted to be circulating in the EA region. The panel is tailored to cover the genetic diversity within the FMDV lineages that circulate in EA countries, including Burundi, Democratic Republic of Congo, Eritrea, Ethiopia, Kenya, Rwanda, Somalia, South Sudan, Tanzania, and Uganda and were selected by phylogenetic analysis of all VP1 sequences available from WRLFMD, the OIE/FAO FMD Reference Laboratory Network, and GenBank databases (Table 3). The panel covers four sets of the serotypes O, A, SAT1, and SAT 2, represented by the lineages O/EA-2, O/EA-3, O/EA-4, A/AFRICA/G-I, A/AFRICA/G-IV, SAT1/I, SAT2/IV, and SAT2/VII, respectively.

## 6. Valency Testing Using the EA FMDV Reference Antigen Panel

Screening of potential vaccine candidates will be carried out by virus neutralisation test (VNT) using sera from cattle vaccinated with each quadrivalent vaccine against the 16 FMDV isolates in the EA FMDV Reference Antigen Panel. At least five cattle sera must be submitted, and these will be tested against the criteria for an acceptable antibody response. At least 70% of individual sera at 21 days post-primary vaccination, or 10 days post-booster vaccination, must meet or exceed the VNT titre set as the threshold, for at least 70% of antigens for each serotype in the panel, and that there must be no evidence of a FMDV-specific antibody in the day 0 sera by non-structural protein (NSP) and VNT antibody testing. In addition, there should be no evidence of FMDV NSP antibodies in the day 21 sera. This is regarded as a “pass” for the valency requirement of the TPP.

## 7. The Vaccine Valency Cut-Off Threshold

Following extensive analysis of available data, the cut-off value for confirmation of a vaccine “pass” by VNT is described on the website of the WRLFMD as follows (recommendation to AgResults on using serological indicators (“valency testing”) of cross protection for FMD vaccines):

Using VNT, an indicator of heterologous cross-protection is considered to be a log_10_ reciprocal titre of 1.5 (cut-off value) after a single dose vaccination with serum collected 21 days later.

Three out of five cattle should have titres at or greater than this level for a pass.

For the AgResults FMD Vaccine Challenge Project, the following information has been published (https://www.galvmed.org/foot-and-mouth-project/resources-potential-competitors/, accessed on 13 September 2021).

For the purposes of the valency test requirement of the Project’s TPP, we will use the WRLFMD, TPI, UK guidance and apply it to the vaccination regime specified by the manufacturer, which may include either a one-dose or two-dose vaccination regime.

Thus, if a log_10_ reciprocal titre of 1.5 or higher is achieved in 3/5 cattle, this will be considered a “pass” for both one-dose and two-dose vaccination sera.

Per the TPP, the vaccine must achieve this “pass” in at least 70% of isolates per serotype set (O, A, SAT1, SAT2)—that is a minimum of three out of four isolates per serotype set.

## 8. Entry into the Cost-Share Phase

Application to the cost-share phase is open to companies that manufacture and supply a suitable FMD vaccine to two or more countries in the region in sufficient quantities to meet market demand in such countries. A manufacturer must submit an application to GALVmed for review by the judging panel, demonstrating that its FMD vaccine has been granted full product registration (defined as approval from the competent regulatory authority to market, sell, and distribute the vaccine in such country in the form of a marketing authorisation, product license, or certificate of registration) in at least two of the target countries in compliance with all the eligibility requirements defined in the competition rules, as assessed by the Project’s judging panel. The vaccine will then be approved as eligible for the cost-share phase of the competition. The application must include supporting documentation sufficient to demonstrate satisfaction of the eligibility criteria, including a copy of the applicable product registration approval documents, all related documentation received from the applicable regulatory authority (including, but not limited to, the summary of product characteristics, product literature, and labelling), all relevant manufacturing authorisations, and such other documentation as is necessary to evidence compliance with GMP requirements together with any other data the competitor determines is relevant to provide evidence that the product as registered meets the requirements of the TPP (Table 1). For clarity, as set forth in the TPP, with respect to testing for vaccine valency, data demonstrating compliance must be generated by a laboratory that (a) has the capabilities to test against the full approved EA FMDV Reference Antigen Panel set forth in the TPP and (b) is neutral, independent, impartial, and conflict free, using batches of product shown to comply with the terms of the product registration. The online application portal was opened on 7 February 2021, and approval of the first vaccine and start of the cost-share phase will happen no sooner than 7 February 2022. All manufacturers and their vaccines are required to adhere to the eligibility requirements throughout the life of the Project to ensure continued quality and efficacy of the vaccine.

## 9. Strengthening FMD Management and Control in Eastern Africa through Public–Private Partnerships in the Vaccine Value Chain

In addition to driving the development and use of high-quality FMD vaccines, the Project has developed a public–private partnership (PPP) framework designed to complement the delivery of the Project’s three objectives. Drawing heavily on the OIE Public–Private Partnerships (PPP) Handbook (https://www.oie.int/app/uploads/2021/03/oie-ppp-handbook-20190419-enint-bd.pdf, accessed on 13 September 2021), the programme will create awareness of the benefits that PPPs could bring to the FMD vaccine value chain (VVC) in EA—from production, purchasing, distribution, delivery, and vaccination to post-vaccination monitoring. Particular aspects of the OIE PPP Handbook are being developed into a practical framework that can help initiate appropriate commitments between partners. The PPP framework addresses the challenges of the FMD VVC and is relevant to the unique FMD control situation in each of the project’s target countries, though it can also be applied more broadly to other livestock VVCs and other geographies. As a notifiable transboundary disease, FMD is highly regulated through the office of the Director of Veterinary Services (DVS), with full mandate for FMD management and control in their country. The livestock VVC (including FMD vaccines) consists of a variety of public and private sector actors, including manufacturers, laboratories, importers, distributors, retailers, vaccinators, veterinarians, veterinary paraprofessionals (VPPs), community animal health workers (CAHWs), and the end-users, who may be commercial and/or smallholder livestock farmers. Each participant in the VVC plays a key role in realising efficient delivery of vaccines and effective management and control of the disease, and where there is improved cooperation between the public and private sectors, this can yield more effective results. It is expected that this framework will serve as a catalyst for future PPP arrangements in the region.

## 10. Scope of the PPP Framework

FMD vaccine procurement in EA is dominated by national governments who operate through tender arrangements and ad hoc direct purchase procedures. Suppliers (manufacturers/distributors) submit price quotes for delivery of products to scheduled government locations and subsequent vaccination campaigns are then carried out by government veterinary officers. None of the currently available FMD vaccines address all EA regional risks, and furthermore, the private sector is unlikely to play a role in vaccine distribution given current market conditions. No multivalent FMD vaccine has been formally registered in the region because of the specialized procurement processes and tenders for access to vaccine. Government purchases are often reactive to outbreaks, leading to unreliable market demand. As such, public sector procurement and distribution efforts do not sufficiently meet the addressable market needs due to constrained budgets and political priorities. Finally, there is limited quality control of FMD vaccines because they are imported through these specialized import processes rather than comprehensive product registration processes.

This current model has several challenges that lead to inefficiencies in FMD control, particularly the sporadic nature of vaccine purchase. Those challenges, described below, are key opportunity areas for PPPs.

## 11. Opportunity Areas

The key PPP opportunity areas identified so far include:

- Partnerships between local government FMD vaccine manufacturers and international FMD vaccine manufacturer on R&D, capacity building, and technical upgrades for vaccine production facilities.
- Partnerships between government and:
  ○ Private vaccine manufacturers to invest in enhanced public sector cold chain capabilities or to outsource cold chain distribution to private sector (private distributors, vets and VPPs).
  ○ Private vaccine manufacturers and distributors for preorder and supply of vaccines and/or to support in demand forecasting.
  ○ Local and international diagnostic laboratories, veterinarians, veterinary paraprofessionals, and livestock producer organisations to build capability and ensure proper coordination, data analysis, and subsequent plans of action following vaccination.
  ○ As the Project progresses, it is expected that interest in both access to high-quality, regionally relevant FMD vaccines and the potential commercial benefits of participating in the VVC will lead to a broad uptake of FMD vaccination and subsequent improvement in the health of the livestock population and the economy of the EA region.

## 12. Conclusions

FMD is highly infectious, present in many regions of the world, and significantly affects the livelihoods of livestock keepers and those in associated industries, particularly in the poorer regions of the world. The highest risk of spread is through the movement of live animals and animal products, and current options for control require adequate disease surveillance, rapid reporting of outbreaks, and implementation of control programmes, usually involving animal movement control, vaccination, and improvements to biosecurity practices. In FMD-free countries, a common method of control is to destroy infected and in-contact animals, followed by rapid disinfection of premises and carrying out of ring vaccination around infected areas. In endemic countries, these are most often not viable options, and so the development of a progressive control pathway by the OIE/FAO, involving improved veterinary services, disease outbreak training, and the use of planned vaccination programmes, has been recognised as the most effective way to combat this most infectious livestock disease. The political will to engage with this process through national governments is rapidly increasing in developing nations, but a major hurdle to success is often having access to suitable, efficacious vaccines. The clustering of FMD viruses into seven virus pools, with three pools covering Europe, the Middle East, and Asia; three pools covering Africa; and one pool covering the Americas, is now enabling a targeted approach to progressive FMD control through the combined activities of OIE and FAO and the regional authorities. The AgResults FMD Vaccine Challenge Project has provided a unique opportunity for the provision of FMD vaccines that will be validated as relevant to the EA region through a valency testing program run by the OIE/FAO FMD Reference Laboratory Network. And through a cost-share component, high-quality, high-potency, quadrivalent FMD vaccines developed through this Project should be available for regional purchase, helping to create greater market stability and a potential reduced cost to end users. It is anticipated that this will lead to an accelerated uptake of vaccination and subsequent reduction in FMD outbreaks in the region, with the end result of improved livelihoods for livestock keepers and their families and improved overall health of the large cattle population in Eastern Africa.

It should be stressed that the use of vaccination alone is not enough for the successful implementation of any FMD control strategy in an endemic setting. Other essential components that must be included are improvements to veterinary services, rapid reporting and identification of outbreak viruses, and increased public and livestock keeper awareness and education on disease spread and the economic benefits of control, along with the necessity for good biosecurity measures to be implemented and maintained on and between farms. Control of FMD in endemic regions is complex and involves many factors working in concert. Through the AgResults FMD Vaccine Challenge Project, it is anticipated that many of the problems reported with previous FMD control efforts can be resolved and that improved access to, and greater confidence in, the use of regionally relevant high-quality FMD vaccines will lead to an overall reduction in the burden of FMD in Eastern Africa and provide economic and health benefits to the livestock keepers, their animals, and the wider community. It is expected that vaccines developed through this initiative may be available from 2023 onwards.

## Figures and Tables

**Table 1:** The target product profile.

	Characteristic	Target Product Profile (TPP)
**1**	**Vaccine** **Valency**	Quadrivalent: A, O, SAT1, SAT2 serotypes that match >70% * (each serotype) of circulating Eastern African foot and mouth disease viruses (FMDV), as defined by the Eastern African FMDV Reference Antigen Panel ** Vaccine testing against the Eastern African FMDV Reference Antigen Panel to be done at an AgResults-approved laboratory ***, which has demonstrated it has (i) the capability to test against the full approved Eastern African FMD panel according to the agreed methodology, (ii) no IP/financial conflict of interest with the vaccine development company, and (iii) accreditation to international standards for the specified testing
**2**	**Host Animal**	Cattle from 3 months of age
**3**	**Efficacy**	Contains a minimum 6PD_50_ per strain per dose For registration: ○ Efficacy requirements as described in the OIE Manual, FMDV chapter 3.1.8 (point 5.3), efficacy testing using challenge virus appropriate to the virus types in the vaccine ○ PD_50_ test to be conducted on monovalent component(s) of the vaccines For batch testing: ○ Indirect potency tests (serology) allowed ○ Batch potency test to be conducted using sera from animals vaccinated with quadrivalent vaccine ○ Prerequisite that to qualify for cost-share payments, each batch considered as ordered/sold is shown to meet this potency requirement
**4**	**Duration of** **Immunity (DoI)**	Minimum 6 months, with maximum of 2 doses
**5**	**Shelf Life**	12 months
**6**	**Differentiating Infected from Vaccinated** **Animals (DIVA)**	(i) Purified vaccine—does not induce antibodies to NSP—or (ii) the response to vaccination in the target species can be differentiated from natural infection in another way (OIE Manual, FMDV chapter 3.1.8, point 5.4)
**7**	**Animal Safety**	Compliant with OIE safety and innocuity standards described in the OIE Manual, FMDV chapter 3.1.8, point 4.1
**8**	**Vial Size**	1 or more vial sizes, at least one of which is to be a maximum of 40 doses per vial to be appropriate for use with smallholder farmers in the region

Footnote: * The 70% refers to the percentage of isolates per serotype for which heterologous titres in post-vaccinal sera are above the quality threshold. ** The Eastern African FMD Reference Antigen Panel includes reference viruses representative of the viruses circulating in 10 countries in Eastern Africa: Burundi, Democratic Republic of Congo, Eritrea, Ethiopia, Kenya, Somalia, South Sudan, Tanzania, Uganda, Rwanda. *** The following laboratories are approved to provide serological testing against the Eastern African FMD panel: 1. World Reference Laboratory for FMD (WRLFMD), Pirbright, UK. 2. OIE Collaborating Centre for Validation, Quality Assessment and Quality Control of Diagnostic Assays and Vaccine for Vesicular Diseases in Europe, Sciensano, Belgium. 3. OIE Reference Laboratory for FMD, ANSES, France.

**Table 2:** FMD viral lineages reported in the competition target countries for the last 5 years.

Target Country	Serotype/Lineage Reported
Burundi	No reports since 2003
Ethiopia	O/EA-3, O/EA-4, A/Africa/G-I, A/Africa/G-VII, A/Africa/G-IV, SAT1/IX, SAT2/XIII, SAT2/VII-^Alx12^, SAT2/VII-^Lib−12^, SAT2/VII-^Ghb−12^
Kenya	O/EA-2, O/EA-4, A/Africa/G-I, SAT1/I, SAT2/IV
Rwanda	SAT2 (reported in 2020)
Tanzania	O/EA-2, A/Africa/G-I, SAT1/I, SAT2/IV
Uganda	O/EA-2, O/EA-4, A/Africa/G-I, SAT1/I, SAT2/IV and SAT2/VII, SAT3 (Buffalo)

**Table 3:** Reported serotypes/lineage in competition target countries aligned with the Eastern Africa FMDV Reference Antigen Panel https://www.wrlfmd.org/node/2096/ (accessed on 13 September 2021).

FMD Reports from Competition Countries (Last 5 Years Approx)							Eastern Africa FMDV Reference Antigen Panel	
Type	Burundi	Ethiopia	Kenya	Rwanda	Tanzania	Uganda	Virus Lineage	Virus Name
**O**	No reports since type O in 2003		O/EA-2		O/EA-2	O/EA-2	O/EA-2	O/KEN/4/2018
		O/EA-3					O/EA-3	O/ETH/4/2015
							O/EA-3	O/ETH/9/2019
		O/EA-4	O/EA-4			O/EA-4	O/EA-4	O/ETH/14/2019
**A**		A/Africa/G-I	A/Africa/G-I		A/Africa/G-I	A/Africa/G-I	A/Africa/G-I	A/ETH/2/2018
							A/Africa/G-I	A/UGA/28/2019
		A/Africa/G-IV					A/Africa/G-IV	A/SUD/9/2018
							A/Africa/G-IV	A/ETH/19/2019
		A-Africa/G-VII						
**SAT1**			SAT1/I		SAT1/I	SAT1/I	SAT1/I	SAT1/TAN/27/2012
							SAT1/I	SAT1/TAN/22/2013
							SAT1/I	SAT1/KEN/10/2013
		SAT1/IX					SAT1/I	SAT1/TAN/22/2014
**SAT2**			SAT2/IV	SAT2 (2020)	SAT2/IV	SAT2/IV	SAT2/IV	SAT2/KEN/19/2017
		SAT2/VII-^Alx12^		Last reports were types O and SAT 2 in 2004 until SAT 2 in 2020		SAT2/VII	SAT2/VII-^Alx12^	SAT2/ETH/16/2015
		SAT2/VII-^Ghb−12^					SAT2/VII-^Ghb12^	SAT2/EGY/1/2018
		SAT2/VII-^Lib−12^					SAT2/VII^-Lib12^	SAT2/ETH/11/2018
		SAT2/XIII						
**SAT3**						SAT3 (Buffalo)		

Further information about the genetic diversity and circulation of these specific viruses can be retrieved from the WRLFMD website and the annual reports of the OIE/FAO FMD Reference Laboratory Network.

## Data Availability

Data available in publicly accessible repositories.
